# Analysing cross-cutting competencies learning in an online entrepreneurship context

**DOI:** 10.1007/s10639-022-11359-z

**Published:** 2022-11-04

**Authors:** Mercedes Marzo-Navarro, Carmen Berné-Manero

**Affiliations:** University of Zaragoza, Zaragoza, Spain

**Keywords:** Cross-cutting competencies, Entrepreneurship, Learning experience, Online commerce, Workshop

## Abstract

Research in teaching innovation encourages leveraging the evolution of digital technologies from using the device to learning with the device, which means a change from using information and communicational technologies to learning and knowledge technologies. Nevertheless, although the feasibility of implementing active and interactive methodologies to improve education is widely recognised, more research is needed to obtain evidence on the subjects and contents with the most significant potential for success. In addition, the most recent literature claims greater attention to the improvement of transversal skills, as they are critical in the student’s professional future. Thus, the main objective of this study is to contribute to the development of immersive learning aimed at improving the cross-cutting skills of university students. Developing a teaching activity where the student acts as an entrepreneur in electronic distribution channels faces the research question. The student-company-university collaboration is the basis for enhancing the transversal skills of the Degree in Marketing and Market Research at the University of Zaragoza (Spain). The student participates in each step of the program as an active agent. The teacher tutors the work teams in each process step, and the Palbin Company provides the necessary technical support. The students give the information to analyse the success of the experience through two surveys -pre and post-workshop, following the methodology used in previous literature. While the activity planning is complex, the students show an excellent mood during the experience. The comparison between expectations and performance offers significant success from the student’s point of view. As a result, the student improves cross-cutting competencies and gains confidence, satisfaction with their learning at university, and professional experience.

## Introduction

Digital media provide solutions to emulate a real-world and even create a sense of user immersion in the digital world (Cummings & Bailenson, 2016). The current digital leadership is a consequence of general acceptation of the immersive technologies in all business sectors and social life (Caputo & Walletzky, 2017), and of the dystopia provoked by the COVID 19 pandemic, which imposed a radical change to face the challenge of substituting presence meetings.

Concerning teaching sector, it is very active in introducing active and innovative methodologies linked with information and communication technologies (ICT). ICT have evolved into learning and knowledge technologies (TAC), taking advantage of learning with the digital device and not just using it (Casasola, 2021). Active methodologies are methods, techniques, and strategies that put the student as the principal agent of their learning and the teacher as its mediator. They are a way of working linked to real-life situations and professional life.

Business community is another ally in learning, mainly matters such as economics, business administration, and marketing. Training students to acquire professional skills is a concern that motivates to promote the link between the company and the university. The professional skills facilitate employment, entrepreneurship, and internal entrepreneurship (corporative entrepreneurship made by employees that develop activities addressed to generate new business for the company, according to Chavarría 2019). Fostering entrepreneurship is an academic challenge, mainly for public institutions, which are usually less focused on practical knowledge. Nevertheless, it is essential to train and prepare the students to enter the labour market and start a business. Mainly due to the current unprecedented increase in the youth unemployment rate. Thus, the triangulation of student-company-university (Berné et al., 2011) is an appropriate context to carry out a project of teaching innovation that focuses on the acquisition of professional competencies.

Moreover, the Spanish Royal Decree 822/2021 considers the cross-cutting competencies development keys. In addition, learning is most efficient and effective when learning by doing. The learning projects must provide approaches to empower students to take direct action in designing and setting up e-shops, for instance, using hands-on learning. The approach has to enable the students to apply to learn, enhance their effectiveness in communication, and develops other competencies (Ngai, 2007).

Although it is widely recognised the viability of implementing the active methodologies and interactive elements to improve the training of students and even teachers, more research and evidence are needed to confirm the effectiveness of matters and contents (see Camarero 2022).

Thus, the main objective of this work is to advance in the development of immersive learning addressed to improve the cross-cutting competencies of university students under the following Proposition (P) and Research Question (RQ):

P. “The student-company-university cooperation, the approximation of actual practice to the classrooms (entrepreneurship), and the leverage of digital resources are main determinants for better academic performance”.

RQ. “To what extent is it effective to implement a programme managed through a digital platform to train university commercial distribution students in cross-cutting competencies and benefit entrepreneurship?“

A practical experience of teaching innovation motivated by student-company-university collaboration serves to address the RQ. It deals with learning cross-cutting competencies of the Grade of Marketing and Market Research, precisely the Commercial Distribution Decisions subject. The Marketing Department of the University of Zaragoza (Spain), 2020–2021, is the academic context in which the students lead antes entrepreneurship project in e-commerce. Due to the pandemic, the university had to approach the academic course through distance classes. The Palbin Company provides a digital platform for electronic commerce and support services.

Technology-based start-ups are an opportunity for the University-Company-State-Society articulation (Benavides-Sánchez et al., 2021). Digital platforms create the need to enhance digital marketing knowledge (Alford & Jones, 2020) and introduce the concept of digital entrepreneurship (Zaheer et al., 2019). In this context, this article presents a training programme designed to allow the students to experiment with a situation of entrepreneurship of a wholesale/ retail business or as sellers of a firm’s products. From a similar format to a Bootcamp, an intensive programme in the short term, the workshop includes technical (digital) learning to develop the web page that supports the online store and practical business organisation and marketing decisions, both strategic and tactic. Students must apply their knowledge in marketing to start a business in a digital channel.

Moreover, learning is most efficient and effective when learning by doing. The providers of products (external companies involved) and the responsible teachers are the ones who evaluate the development and results of the activity. Also, the student judges the interest of the workshop as teaching innovation. Palbin Company assesses the technical quality of the e-shops. The interaction students-company-university is present along every step of the experience.

The method used to assess the utility of the workshop consists of a first questionnaire before starting the workshop, which serves to obtain information on the students’ perception of their current level in every analysed competence and their expectations aroused by the activity. A second questionnaire at the end offers information on the perceptions related to the acquisition of skills through the workshop development. Multivariate methods for independent samples test the comparison between the prior and later information.

Next, this article explains the role of cross-cutting competencies, the procedure that serves to implement the workshop and the collaboration with Palbin Company, the results of the comparison analysis, and the conclusions^1^. The results show the success of the procedure and the experience from the students’ point of view. The students experiment with an entrepreneurship activity. Depending on the entrepreneurship market or business activity (industrial/consumption), they act as an online retailer or a non-commercial intermediary of a particular producer-provider. The students improve their competencies by leveraging the opportunity given by the workshop, the company support, and the continuous tutorial of the teacher.

## The importance of cross-cutting competences

From a bibliometric analysis of educational technology, Chen et al., (2020) conclude that teachers and educators must pay attention to the students in learning and teaching. How they feel and what they can achieve. Moreover, researchers and educators must continuously work on how technology can be better adapted to support education. The authors defend the need to construct learning environments that enable learning anywhere and anytime, taking advantage of the current technology devices.

Nowadays, capabilities as memory are not enough, even useless and non-positive values. The current liquid modernity (Bauman, 2008) needs professionals with traditional skills and others regarding avoiding routines and providing unique values to improve team quality (Boltansky & Chiapello, 2002). They are competencies established, although capable of achieving the success of the individual searching differentiation. These virtues are the trigger of leveraging the specific knowledge of an academic subject, such as marketing management and commercial distribution decision-making. Bauman (2008) points out the importance of evaluating the savoir-être and not only the general or specific knowledge.

These precepts motivate this work to enhance students’ competencies, both the specific decision-making of marketing and commercial distribution and the cross-cutting ones. Competencies are the knowledge, skills, and abilities required for professional success. Knowledge is the intellectual content to be learned; skills are the capacity to apply the knowledge to achieve specific goals and abilities in a professional work environment (Lawson et al., 2014). In this work, the learning of cross-cutting competencies promotes the achievement of specific ones. While the specific competencies linked to any university subject count on consolidated and agreed criteria by the academic staff, cross-cutting competencies must serve as a basis of know-how and essential support of the specific ones. Several theoretical contributions claim the importance of the cross-cutting competencies; Tunning, Careers after Higher Education a European Research Survey (CHEERS), Flexible Professional in the Knowledge Society: New Demands on Higher Education in Europe (REFLEX), Flexible Professional in the Knowledge Society: New Demands on Higher Education in Europe (PROFLEX), OECD’s (Organisation for Economic Co-operation and Development) DeSeCo y LifeComp Report, for instance.

The literature provides structured competencies and ranges of assessments and inferences regarding their various types (Kuz’mina et al., 2020), mainly regarding the knowledge area. The current project selected 19 cross-cutting competencies from the contributions of Mouradian & Huebner (2007) and Chavarría (2019): ethics, empathy, risks, self-demand, autonomy, perseverance, critical-constructive vision, initiative, adaptation, time management, curiosity, planning, organisation, creativity, innovation, and teamwork.

The professor mentioned above, Bauman (2008), wrote about the challenges of education in liquid modernity. Nowadays, the organisations’ commercial structure trends to an increasing deliberated disorganisation. It deals with the “liquid world”, which considers productivity and effectiveness as objectives that must get rid of established knowledge and the rigidity that imposes guidance only by happened and experimentation. There is no perfect organisation, and nowadays, the structures do not maintain their form for enough time to guarantee confidence and long-term responsibility. Therefore, the individual is responsible for providing the best through their abilities and capabilities, which will crystallise in professional competencies.

Specific skills complement cross-cutting, including business development in a natural e-commerce environment and marketing strategies focused on online retail distribution. In this context, both competencies cooperate in managing the knowledge and the student’s motivation for entrepreneurship.

## The procedure

This work focuses on the relationship between companies, universities, and students. However, the two last have the final responsibility for academic success. Thus, during the 14 weeks of the course, the students receive face-to-face classes about commercial distribution theory and practical classes in the computer lab. In the meantime, they get training resources through Moodle platform, information about the role of the involved agents in the practical experience, the keys of teamwork management, and the way to face entrepreneurship.

To explain the practical procedure, the following sections of this manuscript present the involved agents, the meaning of belonging to a team, the entrepreneurship attitude, and the workshop process, as transmitted to the pupils.

### Agents involved

Two previous pilot tests (in 2018–2019 and 2019–2020 courses) with different technology providers partners made it possible to compare them and launch in the 2020–2021 academic year an entrepreneurship and learning workshop on skills related to commercial electronic distribution. This workshop corresponds to the practical classes of the subject Decisions on Commercial Distribution, of the Degree in Marketing. It is possible through the collaboration between Palbin S.L. and the Marketing Department of the University of Zaragoza. 2020–2021 faced the workshop through online classes (Google meet).

Palbin (start-up in 2008; Zaragoza, Spain) is a technology supplier that facilitates the development and start-up of electronic commerce in the cloud through a digital platform. After the previously mentioned experiences, the company’s tool provides more usability and technical resources to the learners than others, according to the student’s point of view. Moreover, Palbin supports the teachers by providing real-time access to every e-shop, which allows a continuous assessment of the work. Further, the students communicate directly with the company staff to solve any technical problem.

The other agent is the teacher, which gives the students permanent tutorial support and resources related to working in groups, entrepreneurship, commercial distribution, and marketing decision-making. First, the students receive a webinar given by the company’s staff about the Palbin platform. Next, as is usual in previous active learning projects, such as Gricar et al., (2005), the students form teams and choose a team leader. The students also receive a significant reduction in prices using the platform that goes longer than the first free month announced on the company’s web. The Moodle platform of the University of Zaragoza facilitates the interaction teachers-students and makes available to them the learning materials.

### Facing the belonging to a team and the entrepreneurship

A team is not just a group. Teamwork develops communicative, influencing, and emotional entrepreneurial skills to practice self-control and manage frustration. Every team usually has a leader capable of motivating them to achieve common goals.

The team members begin training their minds in searching for business ideas viable to a shop online. They have to select one idea from a preliminary list of alternatives. Next, every proposal is discussed with the faculty in class. The selected idea should include an overview of the project, specific considerations, and an organisation plan. Team norms and planning should be established initially, including performance review procedures and team (not just project).

On the other hand, the workshop bears the three primary stages of an entrepreneurship process. The three stages are the generation and selection of ideas, the identification and study of opportunities, the plan and development of the business, searching for partners and resources, implementation, growing up, and consolidation (Chavarría, 2019). In this way, the team works have to take the following fundamental decisions:

i) Search for an original business idea addressed to entrepreneurship through an e-shop. Link this idea with the business objectives.
ii) Think about the business’s mission, vision, and values according to the business objectives. The project must be coherent, original and driven by respect and even sustainable development goals (SDG).
iii) Prepare appropriate storytelling respectful of the project’s authenticity and motivate the goal market by generating curiosity. The importance of the message and its content regards communication decisions.
iv) Analyse the capability of the image and other communication tools based on the image.
v) Take decisions always bearing in mind the achievement of reputation and prestige.
vi) Forecast costs of starting the electronic business, both monetary and non-monetary, to assess the business idea in economic terms.

The students have learned about this matter in the two previous courses and through new practices as extracurricular activities aiming to prompt the entrepreneurial spirit. Nevertheless, the teacher provides them additional training, mainly reviewing aspects such as motivation and leader characteristics, the acquisition of responsibilities, and challenges. They all form the entrepreneurship process, characterised by the pursuit of opportunity, which follows a series of steps by combining knowledge and skills to enable value creation in fulfilling a market need (Pano & Gjika, 2020).

### The workshop process

Firstly, the entire class is divided into work teams, everyone creating and managing their online store. A total of 24 groups result from the division. In addition to the list provided above, they must search for information to analyse the external environment and prepare the entire business plan. Students have to make marketing decisions about the brand name, portfolio, image resources, pricing, commercial communication, social media, and payment methods. Even they have to take into account the offer of post-purchase services. All of them bear in mind the customer orientation of the business. The platform helps its customers (e-shops) decide about promotions and delivery methods differently. Each work team elaborates an Executive Report, two pages maximum, presenting its ideas and developments about the project. The principal value of this document is helpful to the teacher in evaluating the business idea. All of them must be made before building the online shop on the platform. During this first stage of the workshop, the student compliments a survey using Google Forms about their competencies and their expectations about the workshop. The questionnaire has scale questions and open questions.

The second stage regards the development of the seminar in practical classes. At the end of the course, every team presents the new e-shop in class. The students receive the assessment criteria at the beginning of the workshop. Business ideas based on the consecution of SDG and addressed to solve any problem related to the current pandemic scenario are positively valued. A committee formed by the Palbin staff, teachers, and the rest of the teams assess the final results using a template that includes the different valuable criteria.

After the experience, a second survey offers the students’ opinions about performance.

## Methodology of effectiveness analyse

To assess the workshop’s effectiveness, the students compliment a questionnaire before starting the workshop. Students give information about their expectations of participating in the workshop and perceptions of the current level in each competence. A second questionnaire at the end of the course gathers information on the students’ opinions regarding the acquisition and improvement of skills after the workshop development. Moreover, this questionnaire includes open questions about the level of satisfaction with the experience and the potential of the idea to transfer to other courses.

Likert scales of eleven points from 0 to 10, measure the variables. The comparison between the answers of the two questionnaires serves to obtain results about the effectiveness of the experience. Previous research used this method with similar objectives. For instance, Alonso-Martín (2010) assessed the cross-cutting competencies of psychology students in the same way.

The comparative analysis makes it possible to identify the most affected competencies, the changes in attitude and behaviour in the students, and their level of satisfaction with the activity and the method. Data analysis performs using multivariate methods suitable for the comparison of independent samples. The following section presents the results obtained from the information given by the students before and after the workshop development.

The number of students in each sample is 112, the total number of students enrolled in the procedure. Thus, the sample is the entire population under analysis; a total of 20 work teams of 5 individuals and 3 of 4.

## Results

The first result to highlight from the information provided by the first questionnaire is that 100% of the students (112 individuals) who took the online workshop have responded. Achieving this level of participation is a success because the involvement of the student outside of class hours and more to answer surveys is always a challenge. This response informs about the interest and the positive attitude of the students.

**Fig. 1:**
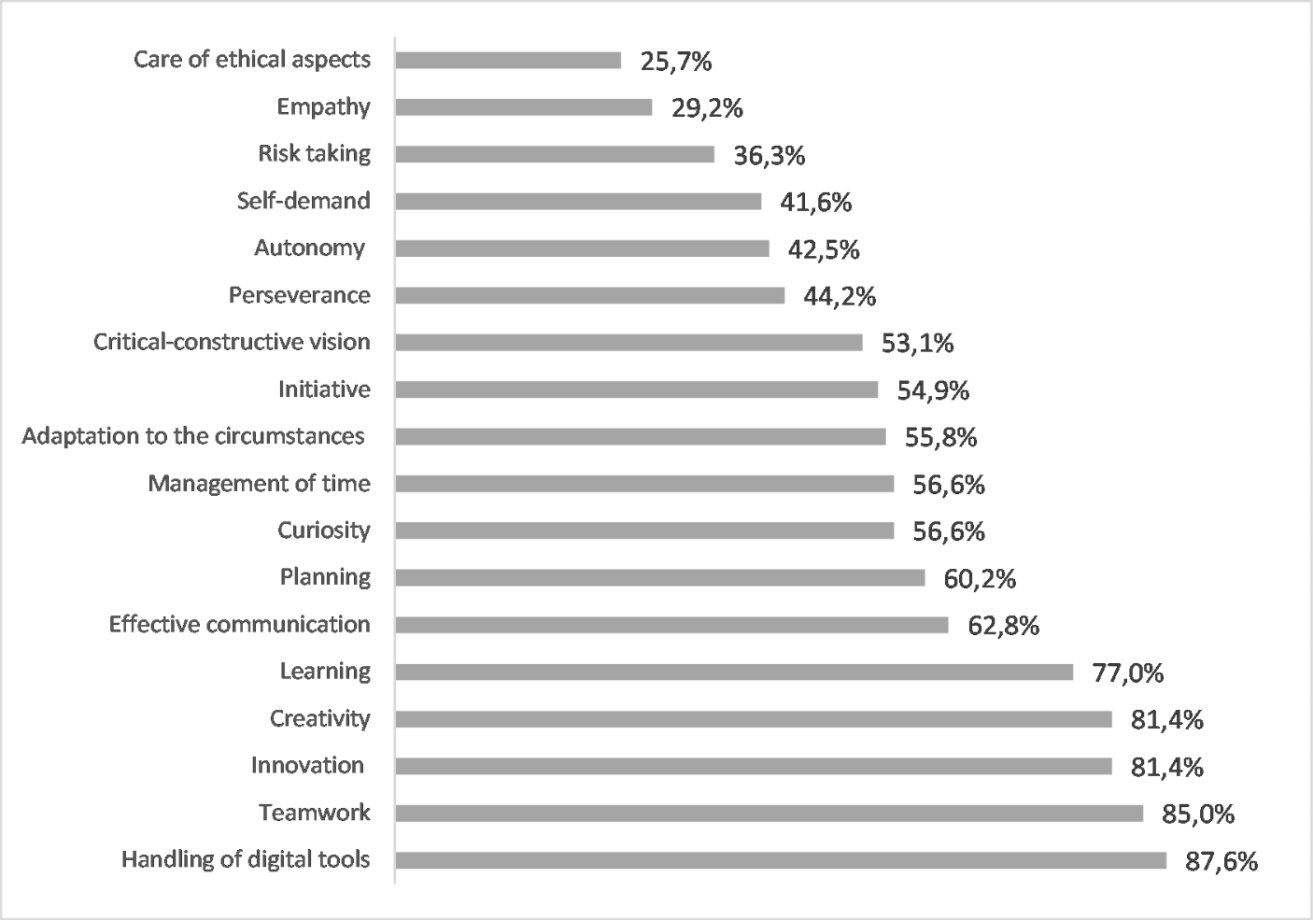
Ranking of competences in percentages. Workshop potential from the students’ point of view

The percentage of affirmative answers to the workshop’s potential to improve each of the 18 competencies considered offers a histogram in which the most mentioned is the use of digital tools (Fig. 1). Since it is a workshop for building a digital business from a web page, its benefit in this skill is evident. The least mentioned competence is related to the care of ethical aspects. Although ethics is antes emphasised topic in class, its relationship to the student workshop may not be very intuitive. The competence for empathy, risk-taking, self-demand, autonomy and perseverance continue in the ranking in a percentage below 50%. These capacities are highly dependent on the individual’s profile or personality characteristics, which they may see as less related to the workshop. However, it should notice that, although their score is low, several respondents consider them as skills promoted by the workshop so that they somehow increase expectations about the activity.

The competencies that exceed 50% of affirmations are 12. The handling of digital tools, the most indicated, adds the critical-constructive vision, initiative, the adaptation to the circumstances (know-how, resilience and overcoming), time management, curiosity, planning, effective communication, learning, creativity, innovation and teamwork. The result shows the primary advantages of a workshop carried out in a team, under teacher direction and supervision, and in a real market context.

Further, Table 1 shows the assessment assigned by the students according to their current level in each competence. A recording of scores from 0 (minimum level) to 10 points (maximum) from low (0–4), sufficient (5,6), notable (7, 8), and outstanding (9, 10) levels serves to clarify the global scores. The result informs that the students have a high opinion about their current level in the analysed competencies. Most of the competencies get a notable score in the opinion of the students. Even handling teamwork and curiosity are competencies considered outstanding for many.

A score under 4 points reflects the individual perception of not having the minimum competence. In this sense, the most affected competence is time management, tied with effective communication. Almost 11% believe that their current level in these two capacities is insufficient. Time management is complex for many. Time is valuable, essential, and something to care about; the passage of time heralds the diminution of opportunities that should be seized and consumed when they present themselves (Bauman, 2008). Therefore, It is optimistic that the students appreciate the importance of leveraging time. In this respect, recognising their low competencies means an excellent motivation for their learning.

Regarding communicative competence, this individual perception among students can draw attention since they belong to Generation Z and, therefore, they are born digital. However, the ability to communicate effectively is something else, and the honesty of these respondents in this regard deserves applause. Digital communication can isolate individuals (Milosevic et al., 2022), mainly in job environments (Toscano & Zappalá, 2020). In a training context, the problem could be similar.

**Table 1:** Percentages of competence assessment (re-cod. from 0 to 10 points)

	LOW (0–4)	SUFFICIENT (5–6)	NOTABLE (7–8)	OUTSTANDING (9–10)
Innovation	8.0	32.7	42.5	16.8
Effective communication	10.6	23.0	44.2	22.1
Empathy	6.2	20.4	40.7	32.7
Handling of digital tools	4.4	19.5	52.2	23.9
Autonomy	3.5	20.4	49.6	26.5
Care of ethical aspects	9.7	24.8	43.4	22.1
Curiosity	3.5	9.7	39.8	46.9
Creativity	3.6	20.5	45.5	30.4
Self-demand	2.7	17.0	46.4	33.9
Perseverance	6.2	22.1	50.4	21.2
Teamwork	1.8	10.6	38.9	48.7
Risk taking	7.1	36.3	43.4	13.3
Management of time	10.6	29.2	38.9	21.2
Initiative	6.3	17.9	44.6	31.3
Learning	0.9	18.8	48.2	32.1
Adaptation to the circumstances	2.7	16.8	48.7	31.9
Planning	6.3	17.1	43.2	33.3
Critical-constructive vision	2.7	25.7	50.4	21.2

The competencies with a level lower than 5 points (C level) may be the ones that most motivate them to carry out the workshop. Combining the competencies qualified as failed with the values that these students assign to the potential of the workshop for each of them, innovation, use of digital tools, creativity, teamwork, time management, learning, planning, and critical view have the most significant potential for improvement by carrying out the activity.

**Fig. 2:**
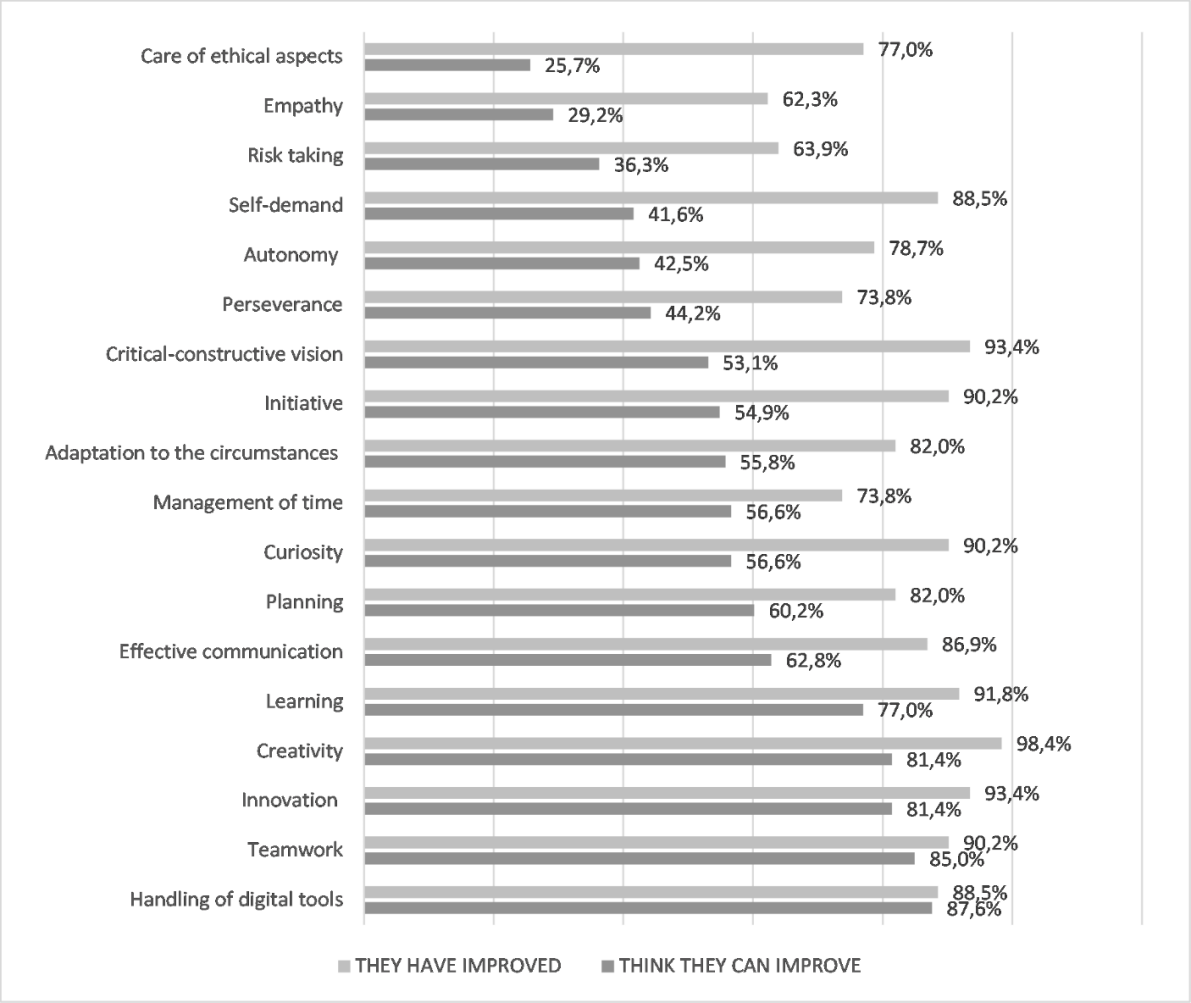
Comparing expectations and perceptions about competencies

Comparing opinions before- and after-workshop shows the great value of the workshop from the students’ point of view. It has a high value in practically all the competencies analysed (Fig. 2). The most significant difference is found in the competence to improve ethical aspects. It was a competition with the lowest perceived potential. The difference with the posterior results is more than 51 points. It is also worth highlighting the differences that show competencies such as self-demand (46.9 points), critical vision (40.3), initiative (35.3), and curiosity (33.6). Digital tools present the closest relationship between the perceptions before and after the development of the workshop (0.9 points). These results also report a considerable change of opinion concerning how the workshop can effectively improve the skills considered so that skills considered little related after the development of the activity get recognition.

An analysis of basic statistics, which includes the mean, standard deviation, median and mode, provides more information on the situation of each competence from the student’s point of view once the workshop finishes. Table 2 collects these data. The competence that offers a higher average statistical value is related to the improvement of creativity, with a deviation of 1.4. It is even higher than the digital tool management variable, whose mean value is 8.2 and the standard deviation is 2.0. The lowest values correspond to empathy, risk-taking and ethics.

Finally, the open questions of the post-activity questionnaire inform that the students think the workshop deserves its implementation in subsequent courses, 93.4% of those surveyed, that the idea can be helpful in other courses, 90.2%, and that it is closer to professional reality than different types of internships, by 91.8%.

Personally, the students expressed their gratitude to the teachers and qualified the idea and the workshop process as the best practice they have had. These data report a high level of satisfaction with the activity and reinforce the previous discourse.

* In dark grey there are the percentages regarding the opinion of the workshop potential in each competence. Light grey colour highlights the perceptions after its development.

**Table 2:** Basic statistics. Post-activity

	Average	Standard deviation	Median	Mode
Innovation	7.3	1.8	8.0	8.0
Effective communication	7.1	2.0	8.0	9.0
Empathy	5.7	2.6	6.0	7.0
Handling digital tools	8.2	2.0	8.0	8.0
Autonomy	7.2	2.1	7.0	9.0
Care of ethical aspects	6.0	2.0	6.0	6.0
Curiosity	7.6	1.9	8.0	7.0
Creativity	8.5	1.4	9.0	9.0
Self-demand	7.3	1.9	7.0	7.0
Perseverance	6.9	1.9	7.0	8.0
Teamwork	7.8	2.1	8.0	8.0
Risk taking	5.9	2.4	6.0	6.0
Time management	6.9	1.9	7.0	8.0
Initiative	7.7	1.8	8.0	8.0
Learning	7.8	1.8	8.0	8.0
Adaptation	7.5	1.8	8.0	8.0
Planning	7.1	2.1	8.0	8.0
Constructive critical vision	7.1	1.9	7.0	6.0

## Conclusion

This study contributes to the research by offering a learning experience that endorses the research proposition and answers the research question. The experience firstly highlights the effectiveness of the collaboration between the company and university to achieve better academic performance. The university resources and business environment benefit the acquisition of competencies and entrepreneurship. The work of Pano & Gjika (2020) offers a similar conclusion.

Second, the procedure facilitates the consecution of the three precepts of Chen et al., (2020). Participating in the method, the students felt that the teachers pay attention to their needs and advances. Their motivation and active participation increased; they also recognised the receiving of global benefits and individual. Therefore, the work made by the three main agents, students, company and teachers contributes to the advance of the scientific knowledge regarding the human and personal approach to the use of technology in higher education. In this sense, the surveys’ results and the basic information of the e-shops inform about the very high student level of satisfaction. They are grateful and recognise the procedure’s strengths to acquire the competencies regarding the subject and advise its use in subsequent courses and even in other subjects. Teachers have the same opinion: other learning contexts may leverage this experience and transfer the workshop’s idea to their teaching objectives.

The process linked to the e-store workshop shows the students the worries of their university and teachers about adopting the available technology to get better performance. The collaboration between practitioners and educators plays an essential role in this sense. The academic objectives and the student’s profile, on the one hand, and the practical knowledge of the companies, on the other, must serve to improve the educational support of the technology. Constructing learning environments such as the one presented here allows learning in the distance and leverages the current digital technologies. Consequently, the method and the results obtained contribute to cover the research lack addressed to evidence the effectiveness of matters and contents in implementing an active and interactive method to improve learning. In this case, by using digital devices in the higher education context.

All in all, collaboration student-company-university, encouraging entrepreneurship, and using available digital resources, improves academic performance. The last responsibility of the procedure and its success is the academic staff. Searching and managing the opportunities and available resources offer practical training in cross-cutting competencies and benefit entrepreneurship. In particular, the e-store workshop is an effective method to enhance the teaching addressed to acquire and enhance cross-cutting competencies in addition to specific and technique ones. Moreover, by implementing the e-commerce workshop described in this article, students get involved in actual business processes. The learning with the device, in a context of learning and knowledge technologies, in this case through a digital platform, allows the creation and implementation of novel business ideas, facilitate to the students experiment as entrepreneurs, learn to test new e-commerce models in a real-life environment, and even to be a businessman exploiting the business once the workshop is over.

This study supports some limitations to consider. The public context in higher education offers many advantages in many senses, but also some limitations. For example, the experience would improve with economic support. The implementation of teaching innovation in a public university depends almost exclusively on the endeavour of the teachers. An active and interactive teaching programme in the learning and knowledge technologies contexts is thus a significant challenge. Government education budgets need to be higher to leverage the digital context advantages. Economic support could facilitate the development of the workshop. More computers per student in the lab, access to other digital devices to complement the learning in the three competencies considered: cross-cutting, specific, and technique, for instance. In addition, the high number of students and teams to manage is a handicap that hinders smooth progress without pressure, reducing effectiveness. Moreover, although online classes are an excellent way to solve circumstances like the pandemic, they could limit the experience. The next course, 2021–2022, will benefit from the face-to-face classes. The personal interaction with the company staff, the classmates, and the teachers could improve the efficiency of the communication and the mechanisms linked to the procedure, avoiding some distance relationship problems.

Future research and teaching experiences in higher education must work together and deepen the development of digital programs capable of improving the quality of training. The Bootcamp format shows strengths in terms of effectiveness, as confirmed by the experience presented here. However, other formats using more sophisticated digital technology are available to those with the necessary resources. Public universities must become aware of the need to invest in teaching innovation to keep up with the current times dominated by digitization, which has a significant impact on training in cross-cutting competencies.

## Data Availability

The datasets generated and analysed during the current study are not publicly available due they corresponds to information obtained by surveys but are available from the corresponding author on reasonable request.

## Abbreviations

**TAC**: learning and knowledge technologies.
**P**: Proposition.
**RQ**: Research Question.
**CHEERS**: Tunning, Careers after Higher Education a European Research Survey.
**REFLEX**: Flexible Professional in the Knowledge Society:New Demands on Higher Education in Europe.
**PROFLEX**: Flexible Professional in the Knowledge Society:New Demands on Higher Education in Europe.
**OECD**: Organisation for Economic Co-operation and Development.

## Footnotes

1 A first version of this research was presented in First IEDIS Conference. It is an international forum regarding employment, digital society and sustainability, organized by the academic institute with the same name. The presentation is available in Conference Proceedings, and there is referenced (Marzo-Navarro & Berne-Manero, 2022) in the Bibliography section of this paper.
